# Driving Type 2 Diabetes Risk Scores into Clinical Practice: Performance Analysis in Hospital Settings

**DOI:** 10.3390/jcm8010107

**Published:** 2019-01-17

**Authors:** Antonio Martinez-Millana, María Argente-Pla, Bernardo Valdivieso Martinez, Vicente Traver Salcedo, Juan Francisco Merino-Torres

**Affiliations:** 1 ITACA, Universitat Politècnica de València, Camino de Vera s/n, 46022 Valencia, Spain; vtraver@itaca.upv.es; 2 Endocrinology and Nutrition Department, University Hospital La Fe, Avinguda de Fernando Abril Martorell, 106, 46026 València, Spain; mariaargentepla@gmail.com (M.A.-P.); merino_jfr@gva.es (J.F.M.-T.); 3 Mixed Research Unit of Endocrinology, Nutrition and Dietetics, La Fe Health Research Institute, Avenida Fernando Abril Martorell, Torre 106 A 7planta, 46026 València, Spain; 4 Unidad Mixta de Reingeniería de Procesos Sociosanitarios, Instituto de Investigación Sanitaria del Hospital Universitario y Politecnico La Fe Bulevar Sur S/N, 46026 Valencia, Spain; valdivieso_ber@gva.es; 5 Home Care and Telemedicine Department, La Fe University and Polytechnic Hospital, 46026 Valencia, Spain

**Keywords:** Risk scores, prediction, T2DM, clinical data, screening

## Abstract

Electronic health records and computational modelling have paved the way for the development of Type 2 Diabetes risk scores to identify subjects at high risk. Unfortunately, few risk scores have been externally validated, and their performance can be compromised when routine clinical data is used. The aim of this study was to assess the performance of well-established risk scores for Type 2 Diabetes using routinely collected clinical data and to quantify their impact on the decision making process of endocrinologists. We tested six risk models that have been validated in external cohorts, as opposed to model development, on electronic health records collected from 2008-2015 from a population of 10,730 subjects. Unavailable or missing data in electronic health records was imputed using an existing validated Bayesian Network. Risk scores were assessed on the basis of statistical performance to differentiate between subjects who developed diabetes and those who did not. Eight endocrinologists provided clinical recommendations based on the risk score output. Due to inaccuracies and discrepancies regarding the exact date of Type 2 Diabetes onset, 76 subjects from the initial population were eligible for the study. Risk scores were useful for identifying subjects who developed diabetes (Framingham risk score yielded a c-statistic of 85%), however, our findings suggest that electronic health records are not prepared to massively use this type of risk scores. Use of a Bayesian Network was key for completion of the risk estimation and did not affect the risk score calculation (*p* > 0.05). Risk score estimation did not have a significant effect on the clinical recommendation except for starting pharmacological treatment (*p* = 0.004) and dietary counselling (*p* = 0.039). Despite their potential use, electronic health records should be carefully analyzed before the massive use of Type 2 Diabetes risk scores for the identification of high-risk subjects, and subsequent targeting of preventive actions.

## 1. Introduction

A booming field in clinical research is the use of mathematical models, also known as prediction models or risk scores, to assess the probability of an individual for developing a disease [1,2]. Such models are based on equations or probabilistic relationships between multiple variables—demographics, laboratory, tests and explorations—that have been collected in a specific context and provide a numeric output that indicates the risk of developing a disease. The implementation of a risk score involves i) model development over a subset of data, ii) model internal validation within a subset of data, and iii) model external validation, to assess performance with new data.

Methodological aspects of the development and validation of risk scores are scientific in nature, with several concerns about model development, internal validation, external validation, and impact evaluation [3]. Type 2 Diabetes Mellitus (T2DM) risk scores aim to precisely identify subjects who may develop the disease in the future, potentially enabling targeting of effective preventive actions [4,5]. Unfortunately, there are few externally validated scores, and moreover, T2DM risk scores are rarely integrated into clinical practice [6]. Shortcomings of external validation studies are attributed to generalization issues (the context of data used for development differs from that of data used in the validation) and lack of data (some variables are not available) [7,8]. Moreover, the quality of hospital-based Electronic Health Records (EHR) is a serious concern, as they contain routinely collected data that might not be as rigorous as data recorded in the context of a clinical study, as risk scores are usually derived from clinical trials [9,10].

The increase in data availability and knowledge from epigenetic studies may improve diagnosis and prognosis of T2DM leading to more effective and efficient management [11,12,13]. Several reasons support the importance of screening for T2DM, including the following: i) the growing prevalence of diabetes in the world [14], ii) the long asymptomatic period before it can be diagnosed [15], iii) undiagnosed rates of TD2M [16], and iv) the fact that newly diagnosed patients already have evidence of microvascular complications [17].

A common limitation of risk scores is data availability, especially when routinely collected clinical data is used. In this context, determining the status of a subject (with/without diabetes) is highly compromised [18]. Most studies reporting T2DM risk scores have identified diabetes cases by using fasting blood glucose measurements and 2-hour glucose values during an oral glucose tolerance test. A few studies use alternative indicators to identify diabetes cases, such as prescription, self-reported outcome, and clinical codes [19]. Moreover, the strategy for missing data imputation is often unclear, and the application of advanced methods is necessary to externally validate the risk model [20]. Routinely collected clinical data may not be as reliable as data collected under a prospective clinical study. As concluded by Riley and colleagues [9], “the quality of e-health records is of particular concern”. A large dataset does not ensure good quality of the records and can in fact mean the opposite. Among the challenges identified, missing data, non-standard clinical diagnostic definitions, and incomplete follow-ups are weaknesses that hamper the adoption of risk scores for T2DM prediction. Moreover, T2DM risk models should be externally validated using data from different settings and populations, because the generalizability outside of the context in which they were designed can be a factor affecting their performance.

We therefore explored the performance and portability of T2DM risk scores in combination with a data imputation strategy based on a Bayesian Network. Six externally validated risk scores were assessed using her, and eight doctors from the endocrinology department of a University Hospital recommended preventive actions based on the risk estimation. Section 2 describes the study design and the clinical scenarios. It also describes the risk scores selected for the study, the missing data imputation strategy, and performance metrics. Section 3 describes the results of the study, and in Section 4 we discuss how these risk scores can be used in clinical practice for improving T2DM diagnosis.

## 2. Material and Methods

### 2.1. Study Design

The study consisted of a single-center randomized study investigating the performance of risk scores for the prediction and detection of T2DM, and a comparison of the effect of the risk score evaluation based on retrospective EHR in University Hospital La Fe of Valencia (Spain). This hospital is the reference clinical setting of La Fe Health Department, a geographical district that covers a population of around 300,000 inhabitants, and it includes two specialties centers and twenty primary care centers. This health department is made up of more than 1100 doctors, 400 residents in training, and around 3800 people in the areas of nursing who provide universal health care services. The EHR of Hospital La Fe has access to data from both primary and specialized care, containing data from the clinical history of the patient and data from hospital admissions.

**Figure 1:**
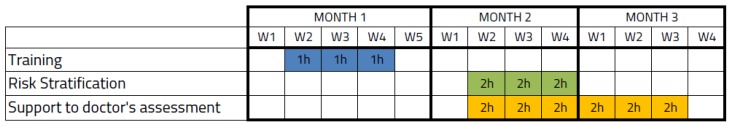
Schedule of the study for the Risk Stratification and the Support to doctor’s assessment.

All de-identified patients who fulfilled inclusion and exclusion criteria entered into a first evaluation. The first evaluation included all patients admitted in the hospital who were initially screened for a T2DM diagnosis. Patients were divided into two groups: cases for patients with confirmed T2DM based on the International Classification of Diseases-9 (ICD-9) code and controls for patients without diagnosis. A web-based system for executing the risk scores [21] was evaluated in the Endocrinology Department of University Hospital La Fe during a continuous 3-month period with the participation of endocrinologists and the head of department who used the tool for 2 hours per session (Figure 1). Three training sessions were planned with the participants prior to utilization of the web tool, which consisted of using the tool in two clinical scenarios (detailed in Section 2.5).

Participants were blindly randomized and analyzed patients from the two groups. Clinical professionals using the tools were recruited according to their role within the Endocrinology Department of University Hospital La Fe after signing the informed consent to participate in the study. The biomedical research ethics committee of the University Hospital La Fe approved, in January 2015, the formal request of data and the study design. 

#### 2.1.1. Inclusion and Exclusion Criteria

The criteria for T2DM diagnosis were based on the American Diabetes Association (ADA) guidelines [22] for fasting blood glucose, HbA1c, and random blood glucose cut-off points. Risk scores were executed on a dataset containing the variables contained in the EHR (Appendix A Table A1). Inclusion criteria were defined as a subject over 45 years old with a confirmed T2DM diagnosis and data availability for five years before the T2DM diagnosis. Subjects were included in the study as controls. The exclusion criteria were defined as T2DM originated by reasons other than ageing and lifestyle (e.g., pancreatic cancer); post-transplantation diabetes mellitus; Type 1 Diabetes Mellitus; patients with a steroid prescription; no data availability for 5 years before T2DM onset; and use of anti-diabetic medication (e.g., metformin in obese adults) for controls. Controls were selected using a propensity score matched-pair procedure with T2DM diagnosis and EHR available for a period of 5 years.

#### 2.1.2. Sample Size Determination

The main outcome of the study was the performance of risk scores to predict and detect T2DM. According to the Spanish incidence rate of diabetes, which is 10.8 cases/1000 person-years [23], and the total adult population covered by University Hospital La Fe Health Department, which is 215,000 subjects, the expected T2DM population was rated as 13,932. After the data extraction process, the study dataset was comprised of 10,730 subjects (77.03%) with data from regular laboratory tests and hospital visits from 2008 to 2015 and a confirmed diagnostic code of T2DM (ICD-9:250.0). According to the extracted data, the incidence rate (cases/person-years) from 2014 to 2015 was 1532. Therefore, assuming a relatively significant improvement of 20% in the prediction and detection of T2DM cases, an experimental ratio of 1:1, and a statistical power of 90% at a 95% confidence level, the minimum sample size was nT = 160 [24].

#### 2.1.3. Retrospective Validation

The main outcome (T2DM diagnosis) was compared to the risk score result (T2DM/not T2DM). Comparisons were based on the following performance metrics:

- Risk score comparison: discrimination and calibration performance of the predictive risk score calculated for every selected case using FINDRISC, ARIC, Framingham, PREDIMED, Cambridge, and San Antonio without calibration.
- Diagnostic power comparison: the proportion of individuals with an HbA1c of 6.0–6.4% or a Fasting Plasma Glucose (FPG) of 110–126 mg/dL thereby being eligible for a preventive intervention, and the proportion of subjects at high risk for the detection model.

Missing data was imputed using a Bayesian Network [25]. The clinical endpoint outcomes of the study were defined as:

- High risk of T2DM cases or T2DM cases.
- A cut-off point for high risk of T2DM cases that would not require blood testing.
- Area Under the Curve (AUC) of Receiver Operating Characteristics (ROC) of the prediction and detection risk tool on the study dataset (also known as c-statistic).

### 2.2. Risk Scores for Type 2 Diabetes Mellitus

A risk score aims to quantify the interaction and relationship between several factors to classify a subject in a binary distribution such as healthy or ill. Such factors may be subject, population, or context-specific, which increases the complexity of validation and generalization. Many predictors or input variables have been proposed over recent decades, but fewer than one quarter have been externally validated [26]. Current ADA guidelines recommend screening for all overweight subjects with BMI ≥25 kg/m^2^ of any age who have one or more TD2M risk factors (hypertension, family history, etc.) [22], whereas the European Association for the Study of Diabetes and the International Diabetes Federation recommend the use of a risk score questionnaire [27].

Many risk scores have been proposed over the last 25 years and they have been compared in systematic reviews with an unclear consensus on which is the best performing risk score [4,28,29]. In their review, Noble et al. [4] analyzed 94 T2DM risk scores tested on 6.88 million participants. From these, the authors judged six risk scores to be the most promising for use in public health practice (Appendix B Table A2) based on the following criteria: 1) Externally validated; 2) Availability of the risk score calculation formulae, and 3) Based on predictors available in the EHR of routine practice. Table 1 reports these six studies that have been externally validated and also includes the PREDIMED study [30] not included in the review mentioned above but relevant for our study. Where a metric was not specified nor available, NS is used. The variables needed for running validated state-of-the-art risk scores, their intercepts, and regression coefficients can be consulted in Appendix A Table A1.

### 2.3. Missing Data Imputation

A recurrent problem when developing and validating risk scores is missing data. To reduce the biases that can occur in a complete case analysis, multiple imputation is frequently used to replace missing values for key risk factors [36]. Multiple imputation [40] is a statistical technique for analyzing incomplete data sets. The issue of missing data is not often reported, and several studies do not report on calibration metrics. A recent research project introduced an algorithm to explore the probabilistic relations between variables comprising T2DM risk factors [25]. This project, which was based on a large longitudinal clinical study [41], provided a Bayesian Network (BN) capable of accurately imputing missing values [42]. The BN contains information regarding the conditional probability relationships among variables, which are weighted in a structure usually represented by a directed acyclic graph. In the present study, missing variables were imputed using this BN model, which is open source

### 2.4. Assessment of Risk Scores

The performance of risk models was assessed by discrimination and calibration metrics [43,44]:

- Discrimination is the ability of the risk prediction model to differentiate between patients who will be diagnosed with diabetes during the observation period from those who will not. Discrimination is quantified by calculating the area under the receiver operating characteristic curve statistic, the Sensitivity (S), the Specificity (Sp), the Positive Predictive Value (PPV), and the Negative Predictive Value (NPV).
- Calibration refers to how closely the risk score outcome agrees with the observed outcome. Calibration of the risk score can be assessed by plotting observed proportions against predicted probabilities; a 45° line denotes perfect calibration. Calibration is quantified by the Hosmer–Lemershow test for the observed and expected events. The *p*-value can be calculated as the right-hand tail probability of the corresponding chi2 distribution for the Hosmer–Lemershow statistic. A *p*-value ≤0.01 indicates poor fitness.

### 2.5. Clinical Scenarios for Risk Assessment

The expected result of the study was the improvement of characterization of T2DM onset and the identification of subjects at risk of developing T2DM.

The BN model permitted data entry for a subset of variables for a patient and estimation of the most probable value for the unspecified variables. This allowed estimation of unspecified variables of interest for better risk characterization such as the 2h-Oral Glucose Tolerance Test (2h-OGTT) and HbA1c. Based on this, we implemented two different clinical scenarios in the screening and risk stratification strategy:

1. Estimate missing variables given available variables measurable with a general practitioner visit and laboratory tests in the EHR and estimate the risk of the subject for developing T2DM.
2. Estimate the 2h-OGTT range given all other available variables (helping the doctor to decide whether a test is needed).

#### Recommendations Based on Expected Risk

According to the ADA guidelines [22], screening for T2DM should be done through an informal assessment of risk factors to guide clinicians on the decision of further standard diagnostic tests, such as HbA1c. At least one annual monitoring is suggested for suspected pre-diabetic stages. Evidence on the effect of lifestyle interventions for the delay and prevention of T2DM comes from the Diabetes Prevention Program (DPP) [45] which demonstrated a significant reduction of T2DM incidence over 3 years. This study was based on a goal-oriented intervention for weight loss and moderate physical activity. Nutrition is also important for reducing the risk of developing T2DM, and data suggest that inclusion of whole grains in the diet could help with this goal [46,47]. Pharmacological interventions including metformin, α-glucosidase inhibitors, and GLP-1 antagonists have been shown to decrease T2DM incidence for pre-diabetic subjects. Finally, self-management and patient empowerment through education and support may be appropriate for maintaining healthy habits and behaviors that may delay or even prevent the development of T2DM. Based on this, we offered nine clinical recommendations:

- Order a 2h-OGTT for this subject.
- Order an HbA1c test for this subject.
- Refer this subject to an endocrinologist.
- Refer this patient to a general practitioner.
- Start pharmacological treatment.
- Prescribe physical activity habits.
- Prescribe dietary habits.
- Counsel on and promote physical activity habits.
- Counsel on and promote healthy dietary habits.

Depending on the estimated risk, the endocrinologist had to select either no recommendation or any of the aforementioned recommendations for each subject based on the surrounding conditions, context, and expertise.

## 3. Results

**Figure 2:**
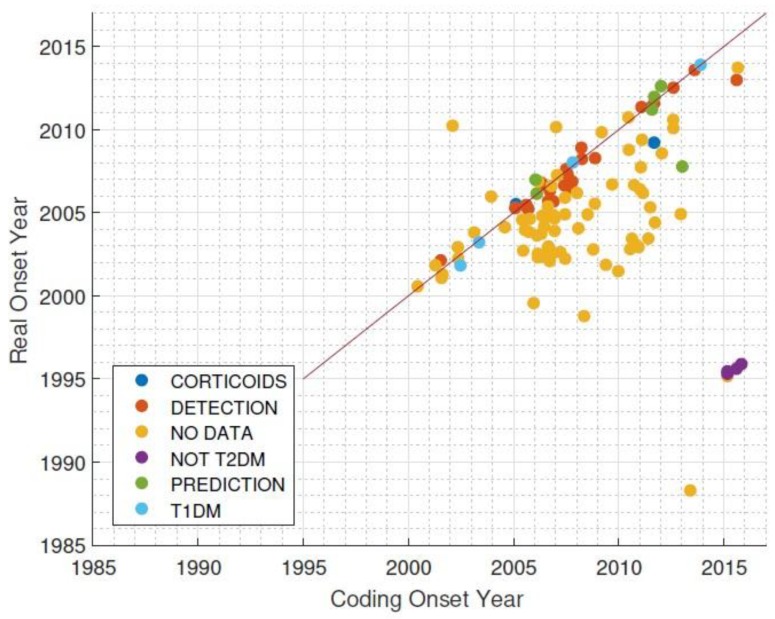
Difference between coding onset year and real onset year for Type 2 Diabetes Mellitus diagnoses. T1DM = Type 1 Diabetes Mellitus.

A total of 159 subjects meeting the inclusion criteria and not meeting the exclusion criteria were included in the study. After conducting an individual analysis of the hospital records for each patient in the cohort, supposedly diagnosed between 2014 and 2015, we concluded that the ICD-9 codifications for T2DM were erroneous, and the majority of the patients developed diabetes several years earlier than expected (Figure 2).

After analyzing the 159 subjects, *n* = 76 patients were eligible and were recorded on the system database. The low incidence rate was due to a lack of quality in the disease coding of the electronic medical record (ICD-9). Case-by-case revision of patients was done according to established criteria [22]. The main limitation was finding patients who had developed diabetes and had clinical records of at least five years before the real disease onset. The prediction span of risk scores is shown in Appendix B Table A2. This fact was a key issue in locating T2DM patients and the availability of records that could fulfil the criteria defined in the study.

### 3.1. Evaluation of Prediction Risk Scores for T2DM Performance

A total of n_P_ = 25 subjects (13 controls and 12 cases of T2DM) were recorded to assess both discrimination and calibration. Independence of variables was assessed by a two-sided t-Student test at IC = 95%. All variables were independently distributed with respect to the patient group (T2DM/no-T2DM), except for diastolic blood pressure, which is not identified as a predictor in any of the considered risk scores.

**Figure 3:**
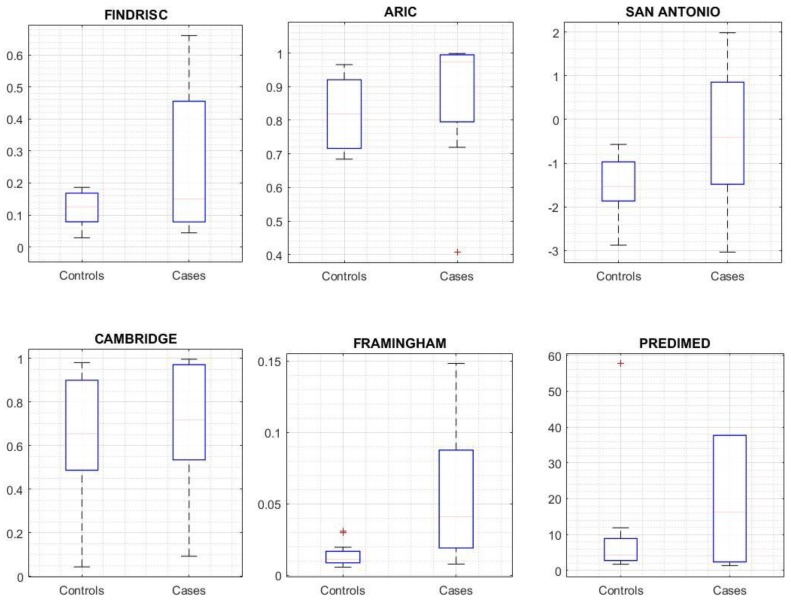
Risk Score outcome comparison between cases and controls.

**Figure 4:**
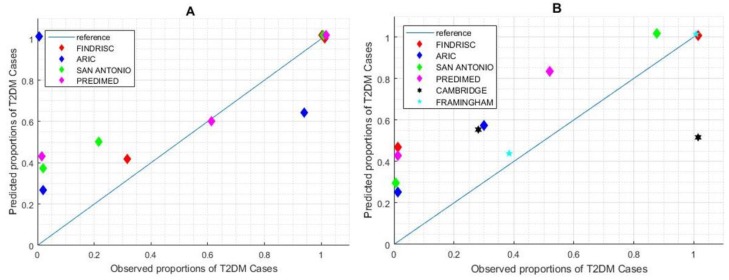
Calibration performance of risk scores with suggested and calculated cut-off points. (**A**) Calibration plot for suggested cut-off. (**B**) Calibration plot for re-calculated cut-off. Cambridge and Framingham scores do not suggest cut-off points, so the performance descriptors are not applicable in chart (**A**)**.**

After the execution of the selected risk scores, the distribution of the outcome was analyzed with respect to the group (Figure 3). Only Framingham (*p* = 0.005), San Antonio (*p* = 0.018), and FINDRISC (*p* = 0.048) achieved a significant difference for the observed outcome. Table 2 shows the discrimination and calibration performance for the recalculated cut-off points (those that maximize the AUC ROC), and Figure 4 shows the calibration plot for each risk score. According to these outcomes, the Framingham risk score model performs better at predicting subjects development of T2DM using a threshold of 0.034.

### 3.2. Support on T2DM Screening

**Figure 5:**
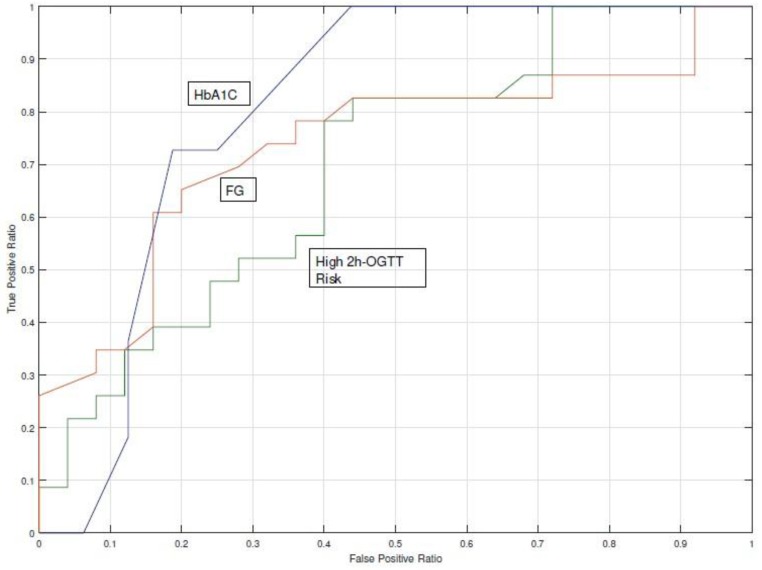
Comparison of the c-statistic (AUC Receiver Operating Characteristics (ROC) curve) for the 2h-OGTT high-risk probability and the two gold standard procedures.Fasting Glucose (FG) and HbA1c).

Detection of T2DM cases was done using the Bayesian Network model [25] on the nD = 48 population (23 cases and 25 controls). This model calculates the probability of having a low (<140 mg/dL), medium (140–199 mg/dL), or high (>200 mg/dL) 2h-OGTT test, which is the standard ADA criteria. The BN model provided a probability for each range, for instance: 80% LOW, 15% MEDIUM, and 5% HIGH. The purpose was therefore to find the thresholds for these probabilities that performed a better classification among subjects who developed T2DM and those who did not. We compared the classification performance yielded by the three detection strategies on the analysis of HbA1c (cut-off 6.5%), fasting glucose (cut-off 126 mg/dL), and the estimated high 2h-OGTT risk. The results were AUC = 0.81 for HbA1c, AUC = 0.74 for fasting glucose, and AUC = 0.69 for high 2h-OGTT risk. Figure 5 shows the ROC curve diagram.

### 3.3. Missing Data Influence on Risk Score Outcome

#### 3.3.1. Prediction Analysis

All risk models needed the input predictors to estimate the risk of developing T2DM. If a variable was missing the risk score equation could not be used (Table 3 shows missing data rate per variable). To overcome this recurrent problem in EHR we imputed missing data using a Bayesian Network specifically designed for T2DM [42]. 

In this analysis we focused on the influence of imputed variables—estimation of missing variables—on the risk output. Our analysis confirmed that the percentage of missing data was not a factor related to the risk estimation (*p* > 0.05). Only the Framingham risk score was slightly affected by the number of imputed input variables (*p* = 0.049). 

#### 3.3.2. Detection Analysis

The ADA guidelines define diagnostic cut-off points for HbA1c, fasting glucose, and 2h-OGTT and, of these, the first and the third may not be present in electronic records unless a doctor specifically ordered the particular test. Moreover, the 2h-OGTT is less available than the HbA1c, as the latter can be determined in a regular laboratory test and the former requires a 2-hour-long test. For the data set used in this study, missing HbA1c accounted for 54% of the cases, whereas missing fasting glucose accounted for only 6% (Table 4). The risk estimated for a high 2h-OGTT was available for all patients by means of the BN missing data estimator [42].

The risk estimated for a high 2h-OGTT underperformed when compared with HbA1c and fasting glucose (Figure 5). The two-sided *t*-Student test for fasting glucose distributions rejected the null hypothesis that HbA1c and fasting glucose from cases and controls-observations had the same distribution (*p* < 0.05), whereas the null hypothesis was not rejected for the high 2h-OGTT risk (*p* = 0.899). The AUC ROC achieved by the fasting glucose indicator with a cut-off point of 126 mg/dL was 77% and for the high 2h-OGTT risk it was 55%. These analyses confirmed the results obtained in the detection model analysis, as the 2h-OGTT estimator does not perform a better classification when HbA1c or fasting glucose are available.

### 3.4. Clinical Advice for High-Risk Subjects

The eight endocrinologists enrolled in the study (Table 5) assigned clinical recommendations to subjects based on the estimated risk.

The system calculated the risk of developing T2DM for each subject and presented the estimation through a web interface to the clinician, who had to make an assessment based on the available clinical data, the inferred parameters with the BN model, and the estimated risk. Based on this assessment, the clinician had the option of selecting one of the nine recommendations described in the methods section.

Table 6 shows the selected recommendations classified for the estimated risk (Risk Outcome). In total, 19 out of 23 cases (82.6%) identified as high-risk (true positives) were assigned to pursue an HbA1c analysis, which is the most specific test for discriminating the diagnosis, whereas only 13 out of 23 (56.2%) of the real cases were assigned to do the test. 

## 4. Discussion

### 4.1. Advancing the Prediction and Diagnosis of T2DM

This study assessed six externally validated risk scores for the prediction of T2DM: FINDRISC [19], ARIC [32], San Antonio [34], Cambridge [37], Framingham [39], and PREDIMED [30].

All these models achieved c-statistic values ranging from 66% to 85% both in internal and external validation studies in the literature. In our study, the Framingham risk score yields an area under the ROC curve of 87.5%, which is an improvement on previous studies, whereas the rest of the risk scores perform within the aforementioned range. Among the analyzed risk scores, we found a high variability in the number of parameters used (predictors) and their relative weight (coefficients).

The results of the application of these models in clinical settings confirms their usefulness to discriminate high-risk T2DM patients. Nevertheless, data quality is a shortcoming that affects the scalability of this type of solution for high-risk subject identification. EHR data may not be of sufficient quality to develop T2DM risk scores. The main pitfall of EHR in our study was the lack of consistency between the coded T2DM onset (first time ICD-9 250.0 code was registered) and the actual T2DM onset of the patients (when the patient was diagnosed), which can bias the relative weight of the predictors towards outcome discrimination. We therefore suggest the creation of a specific code for the registration of T2DM onset, different from the regular ICD label, that refers to “diabetes mellitus”, and the development of advanced models to identify the exact T2DM date based on retrospective EHR datasets.

Currently, several healthcare services lack a homogeneous program for the screening and prevention of T2DM. Clinical guidelines for T2DM do not propose methods to identify people at high risk using the risk scores in an automated manner. Our study introduced the concept of proactive search that allowed for the identification of high-risk populations. We propose a screening strategy based on the estimation of missing parameters with a Bayesian Network [25] to impute missing parameters, such as the 2h-OGTT. Although 2h-OGTT is considered as the gold standard to establish a diagnosis of pre-diabetes or diabetes, when using the estimated data to calculate the AUC, we found that estimated 2h-OGTT had the lowest AUC among the screening tests (AUC = 0.81 for HbA1c, AUC = 0.74 for fasting glucose, and AUC = 0.69 for high 2h-OGTT estimation), which could be caused by an inaccuracy of the estimation model or a different scenario of probabilities. Thus, limitations of the missing data estimation should be handled beforehand.

Our findings suggest that the integration of the risk score in the clinical process in combination with subject-oriented lifestyle intervention could reduce the incidence of T2DM. This approach is limited because of the lack of assigned resources to perform targeted screening and lifestyle intervention in most clinical settings. Our work represents a relevant case study to illustrate the viability of such a screening strategy. 

Compared with FPG and HbA1c cut-off points, the 2-h OGTT value diagnoses more people with diabetes [22]. The implementation of an accurate model for estimating the risk related to a given 2h-OGTT will drive the implementation of cost-effective precise interventions to delay or even prevent the onset of T2DM. 

### 4.2. Prediction and Detection of T2DM in Clinical Settings

The imputation of missing data was key to the assessment of the performance of the risk scores using available EHR data. Data quality and availability is a critical issue that should be examined by the Information Technology service of a hospital in order to clean and ensure the consistency of the records prior to risk evaluation. Based on this, it is feasible to define a proactive screening strategy based on risk scores and models, which have shown acceptably accurate results.

The American Diabetes Association recommends the screening of all adults over 45 years old and of patients with BMI ≥25 kg/m^2^. In the case of a negative test result—no diabetes or pre-diabetic states are diagnosed—the recommendation is to screen every year. By adding risk scores [39] and imputation models [25], we can propose a more proactive screening strategy in which a process of selective screening could be done using available data, without the need for new laboratory tests. 

Risk scores have been tested in an endocrinology service, giving experts in diabetes the possibility to assess the tools in their clinical practice. However, the risk models presented the problem of data quality. From our findings we draw the following recommendations: 1) Proactive searches can be used to select high-risk populations. Based on discussions with medical experts and the real users of these tools, the proposed tool is a novel opportunity to identify new cases of TD2M using existing data. 2) Interdepartmental coordination. The use of the tools generates new clinical processes that were not previously possible in the health center or hospital. Potential barriers that should be managed are data access and the allocation of sufficient resources for all the four actions of the screening tools: proactive searching, risk stratification, case revision, and actions of screening and prevention. These need to be addressed in order to consolidate the process in the context of real clinical practice. 3) The process should be as automatic as possible. Risk stratification should be done automatically in the background and integrated into the health care records as additional clinical information to be presented in the patient health records. 4) Quality of the data. In order to successfully implement the tools in health care settings, it is strongly recommended to assess the quality of the data and verify possible missing data along with errors in the codifications and similar issues. Risk scores have the potential of fitting into the current T2DM prevention and detection campaigns [6]. To this end, the assessment of the effectiveness of a public health campaign, clinical protocol, or medical technology—drug, combination of drugs, recommendations or monitoring system—would be directly driven for enhancing the success odds of specific high-risk subjects.

In the analyzed data, the Framingham risk score achieved an area under the ROC curve of 87.5%, which is an improvement on previous studies. This may be because the number of predictors is significantly higher than the other risk scores (10 predictors instead of 5-6-8 predictors) and the coefficients for predictors directly related to a higher T2DM onset probability (age, BMI, and waist circumference) have a relative increased weight. Categorization of continuous variables such as fasting glucose, waist circumference, and BMI could compromise the performance of the model, which has been reported in regression models for other diseases [48].

Clinicians are likely to choose pharmacological preventive interventions and healthy lifestyle recommendations for high-risk subjects whereas the recommendations decrease for lower-risk subjects.

Despite the shortcomings in data completeness and diagnosis timestamps, we have demonstrated that risk scores can fill the gap within prevention strategies. Guillies and colleagues [49] concluded that subjects at high risk of developing T2DM who were assigned with an intervention reverted the development of the disease in approximately 20 cases per 100 person-years. The use of proactive screening strategies such as the strategy presented in this study could bridge the gap and identify the 80 remaining cases earlier, enabling closer follow-up and reducing the burden of the disease with individually-targeted secondary-prevention based on patient empowerment, adequate diet, moderate physical activity, and prevention of complications.

### 4.3. Limitations of the Study

Data quality is a major shortcoming that affects the scalability of actions aiming to identify high-risk subjects, because both inappropriate timestamps for ICD-9 coding and incomplete EHR were frequent. The imputation of missing data was based on a validated Bayesian Network tool specifically developed for T2DM subjects, which may influence the estimation of missing parameters for the healthy cohort. The sample size of our study is a critical point. Although the initial data sample was 10,730 subjects, the aforementioned issues found in the EHR limited the validation study. Nevertheless, all cases were supervised, ensuring T2DM detection and preventing prior miss-classification. The selection of risk scores may be expanded with other risk scores and new publications investigating prediction and detection of T2DM [50], especially because more risk scores are continuously being designed to be used when routinely collected healthcare data are available.

## 5. Conclusions

The integration of existing prediction and detection risk scores for T2DM based on EHR enables the detection of high-risk cases, whereas detection models underperform with respect to state-of-the-art clinical guidelines.

EHR are not prepared to execute predictive risk scores due to deficiencies in the quality of the data. The main shortcoming is the inaccuracy of the disease-specific coding timestamp, which is different to the actual onset date. The second shortcoming is the lack of data (missing predictors) needed to execute prediction and detection risk scores.

After recalibration, the Framingham risk score properly classified a significant cohort of the study sample as diabetic (AUC = 85%), enabling targeted preventive treatment for delaying the onset of T2DM. Without recalibration and use of the suggested cut-off points, only FINDRISC (AUC = 69%) and San Antonio (AUC = 73%) provided an acceptable classification accuracy.

The risk of having a positive 2h-OGTT underperformed (AUC = 69%) with respect to the fasting glucose test (AUC = 74%) and the HbA1c test (AUC = 81%). Fasting glucose data availability was close to 100%, which suggests that support for a clinician to decide if a 2h-OGTT is needed can be based on this indicator, without the need of having a model to simulate the most probable outcome of a 2h-OGTT. HbA1c data availability was below 50%, which suggests that in this case a predictive tool could be useful. Clinicians are likely to choose among pharmacological preventive interventions and healthy lifestyle recommendations for high-risk subjects, whereas fewer recommendations are made for low-risk subjects.

## Figures and Tables

**Table 1:** Sample size, threshold, and discrimination performance of the externally validated risk models selected for the assessment.

Risk Score	Sample Size	Incident Cases of T2DM	Cut-Off Point	S	Sp	PPV	NPV	AUC
FINDRISC Internal [19]	4586	182	≥9	0.78	0.77	0.13	0.99	0.85
FINDRISC External [31]	18,301	844	≥7	0.76	0.63	0.11	NA	0.76
ARIC Internal [32]	7915	1292	≥0.18	0.67	0.77	0.36	0.92	0.80
ARIC External [33]	5329	446	NS	NS	NS	NS	NS	0.84 *
San Antonio Internal [34]	2903	275	NA	NS	NS	NS	NS	0.84
San Antonio External [35]	2395	124	>0.0065	0.75	0.72	0.119	NS	0.83 *
QDScore Internal [36]	3,773,585	115,616	NS	NS	NS	NS	NS	0.83 men 0.85 women
QDScore External [36]	2,396,392	72,986	NS	NS	NS	NS	NS	0.80 men 0.81 women
Cambridge Internal [37]	24,495	323	>0.37	0.55	0.80	NS	NS	0.75
Cambridge External [38]	5135	302	>0.37	NS	NS	NS	NS	0.72
PREDIMED Internal [30]	1381	155	≥6	0.72	0.72	0.25	0.95	0.78
PREDIMED External [30]	552	124	≥6	0.85	0.26	0.25	0.86	0.66
Framingham Internal ** [39]	3140	160	NS	NS	NS	NS	NS	0.84
Framingham External ** [33]	5329	446	NS	NS	NS	NS	NS	0.83 *

* Indicates recalibration. ** Specific model for the prediction of Type 2 Diabetes Mellitus (T2DM) derived from the Framingham Offspring Study. S = Sensitivity; Sp = Specificity; PPV = Positive Predictive Value; NPV = Negative Predictive Value; AUC = Area Under the Curve; and NS = metric not specified.

**Table 2:** Discrimination and calibration of the risk models for recalculated cut-off points

	S	Sp	PPV	NPV	AUC	Cut-off	HL Score	*p*-Value
**FINDRISC**	0.38	1	1	0.6	0.69	0.180	0.003	0.043
**ARIC**	0.53	1	1	0.67	0.73	0.821	0.271	0.397
**SAN ANT**	0.61	1	1	0.71	0.76	0.065	0.018	0.107
**PREDIMED**	0.54	0.91	0.83	0.57	0.66	16.297	0.049	0.175
**CAMBRIDGE**	0.76	0.33	0.55	0.57	0.53	0.345	0.288	0.408
**FRAMINGHAM**	0.85	0.83	0.84	0.83	0.875	0.034	<0.001	0.020

S = Sensitivity; Sp = Specificity; PPV = Positive Predictive Value; NPV = Negative Predictive Value; AUC = Area Under the Curve; HL: Hosmer–Lemershow.

**Table 3:** Descriptive distribution, dependency analysis, and missing data rate for Cases and Controls of the prediction analysis.

VARIABLE	CONTROLS (*n* = 13)		CASES (*n* = 12)		*p* Value	MISSING DATA (%)
Gender	4 M/9 F		5 M/7 F			
	*Mean*	*SD*	*Mean*	*SD*		
Age	65.76	8.20	59.41	9.28	0.082	0
Body Mass Index	28.78	5.20	32.16	8.46	0.433	56
Waist	98.66	5.13	92.00	0.00	0.377	84
Systolic Blood Pressure	130.00	12.94	136.67	21.82	0.451	36
Diastolic Blood Pressure	75.30	9.86	89.83	12.30	0.020	36
Pulse	70.85	8.78	74.00	12.20	0.613	52
Cholesterol	198.31	48.62	208.50	31.53	0.544	0
Triglyceride	149.23	60.63	175.75	61.96	0.290	0
High-Density Lipoprotein (HDL)	45.58	17.16	49.11	13.67	0.618	16
Fasting Glucose	101.55	12.34	98.27	10.51	0.510	12
HbA1C	5.89	0.37	5.58	0.40	0.132	32

**Table 4:** Descriptive distribution, dependency analysis, and missing data rate for Cases and Controls of the detection.

VARIABLE	CONTROLS (*n* = 25)		CASES (*n* = 23)		*p* Value	MISSING DATA (%)
Gender	12 M/13 F		13 M/10 F			
	*Mean*	*SD*	*Mean*	*SD*		
Age	61.6	8.98	62.35	11.18	0.800	0.00
Body Mass Index	29.22	6.14	32.13	7.87	0.319	45.80
Waist	96	6.10	115	24.95	0.262	85.40
Systolic Blood Pressure	135.41	18.514	128	16.749	0.237	31.25
Diastolic Blood Pressure	82.41	12.76	79.5	9.07	0.020	36.00
Pulse	71.25	10.83	81.92	12.62	0.030	45.83
Cholesterol	204.76	41.43	203.23	41.75	0.900	2.08
Triglyceride	177.52	94.29	195.9	68.36	0.290	0.00
HDL	45.58	17.16	49.11	13.67	0.643	4.16
Fasting Glucose	100.82	11.083	108.13	8.95	<0.05	6.00
HbA1C	5.75	0.41	6.17	0.19	<0.05	54.00

**Table 5:** Endocrinologists evaluating the two clinical scenarios. Information Technology (IT).

**Gender**	Male(2)/Female (6)	
**Age (Years)**	42 ± 13	
**Professional Experience (years)**	14 ± 10	
**IT Literacy (Self-reported)**	High = 3; Medium = 3; Low = 2	
**Patients assisted (number of)**	Overall	319.33 ± 247.66
	TD2M Patients	127.44 ± 75.22
	High Risk of developing T2DM	48.00 ± 33.79

**Table 6:** Number of recommendations for each subject according to the risk outcome. Low and high risk discrimination is done at the recommended cut-off point.

Recommendation	Risk Outcome		Statistical Analysis	
	LOW RISK	HIGH RISK	*p*	Chi2
Order an 2h-OGTT for this patient	4	6	0.654	0.20
Order an HbA1c test for this patient	15	19	0.466	0.52
Refer to General endocrinologist	1	2	-	-
Refer to General Practitioner	11	12	-	-
Start Pharmacological Treatment	1	8	0.004	8.00
Start Dietary Habits	5	12	0.039	4.23
Start Moderate Physical Activity Habits	6	11	0.170	1.88
Counsel about healthy lifestyle	15	11	0.405	0.69
Counsel about diet, physical activity, and weight control	6	11	0.170	1.88

## Appendix A

Externally validated list of Risk Scores and description of the derivation sample population characteristics, mathematical approach, and criteria for type 2 diabetes diagnoses.

**Table A1:** Externally validated list of Risk Scores and description of the derivation sample population characteristics, mathematical approach, and criteria for type 2 diabetes diagnoses.

	FINDRISC	ARIC	San Antonio	PREDIMED	Framingham	CAMBRIDGE
**Intercept**	−5.51	−9.981	−13.415		−18.607	−6.322
**Age (years)**	45–54: 0.63 55–64: 0.89	0.0173	0.028		50–64: −0.010 ≥65: −0.2107	0.063
**Gender**			Female: 0.661		Male: 0.4308	Female: −0.879
**Ethnicity**		African-American: 0.443	Hispanic: 0.44			
**Anti-hypertensive medication**	0.71			0.838	0.336	1.222
**Fasting glucose (mg/dL)**	>110: 2.14 *	0.088	0.079	≥100: 1.929	0.1398	
**BMI** **(kg/m^2^)**	25–30: 0.17 >30: 1.10		0.070	≥27: 0.315	0.03922	25–27.49: 0.6 9927.50–30: 1.970 >30: 2.518
**HDL**		0.0012	0.039		−0.0488	
**Triglyceride**		0.00271			≥150: 0.405	
**Blood pressure (mmHg)**		Systolic: 0.0111	Systolic: 0.018	*130/85 ****	Systolic: 0.001	
**Family history of diabetes**		0.498	0.481	0.506	0.4383	0.728 **
**Smoker**				0.547		0.855 **
**Alcohol**				0.427		
**Waist circumference** **(cm)**	Men 94–102 Women 80–88 0.86 Men ≥102 Women ≥ 88 1.35	0.0273			0.0488	
**Height (cm)**		0.0326				

* Refers to any hipoglycemia. ** The original model foresees two more categories not available in the study dataset. *** The model calculates high blood pressure as an analysis of Systolic and Diastolic blood pressure or anti-hypertensive medication prescription, only one of the predictors is used.

## Appendix B

Externally validated list of Risk Scores and description of the derivation sample population characteristics, mathematical approach, and criteria for type 2 diabetes diagnoses. World Health Organization (WHO); 2h- Plasgma Glucose (2h-PG); American Diabetes Association (ADA);Electronic Health Record (EHR).

**Table A2:** Externally validated list of Risk Scores and description of the derivation sample population characteristics, mathematical approach, and criteria for type 2 diabetes diagnoses.

Risk Score Name and Validation Study	Population Characteristics for Internal Validation	Population Characteristics for External Validation	Mathematical Model	T2DM Diagnosis Criteria
FINDRISC [1,2]	NS	North European, Dutch, Australian, African	Logistic regression	WHO (FPG or 2h-PG)
	Ages: 35–64	Ages: 35.2–71		
	Follow-up: 5 years	Follow-up: 5 Years		
ARIC [3,4]	United States Communities (85% white; 15% African-American)	United States Communities	Logistic regression	WHO criteria or clinical diagnosis or diabetic treatment
	Ages: 45–64	Ages: 45–84		
	Follow-up: 9 years	Follow-up: 4.75 years		
San Antonio Internal [5]	Mexican-Americans and Random Sample	Finland and Sweden	Linear regression	ADA criteria (FPG or 2h-PG only)
	Ages: NS	Ages: 44–55		
	Follow-up: 7.5 years	Follow-up: 7.5 years		
QDScore Internal [7,8]	Caucasian	Caucasian (93%) and other ethnic groups	Proportional hazards model, multiple imputation	Diagnosis read code for diabetes in EHR
	Ages: 25–79	Ages: 25–79		
	Retrospective (15 years) Qresearch Data Base	Retrospective (15 years) THIN DataBase		
Cambridge Internal [9,10]	UK population	UK population	Logistic regression	Diagnostic Code or diabetic medication
	Ages: 40–79	Ages: 35–55		
	Follow-up: 5 years	Retrospective data base (11.7 years)		
PREDIMED Internal [11]	Spanish Caucasian	Spanish Caucasian (High Risk)	Multivariate Cox regression	ADA criteria (FPG or 2h-PG only)
	Ages: 55–80	Ages: 45–75		
	Follow-up: 3.8 years	Follow-up: 4.2 years		
Framingham Internal [4,12]	Caucasian	Caucasian, African-American, Hispanic, and Chinese-American	Logistic regression	ADA criteria (FPG or 2h-PG only)
	Ages: 44.2–63.9	Ages: 45–84		
	Follow-up: 7 years	Follow-up: 4.75 years		

World Health Organization (WHO); 2h- Plasgma Glucose (2h-PG); American Diabetes Association (ADA); Electronic Health Record (EHR).
